# Impact of implementing primary care-based medication for opioid use disorder on provider and staff perceptions

**DOI:** 10.1093/fampra/cmae044

**Published:** 2024-09-23

**Authors:** Sara Mazzarelli, Audrey L Blewer, Truls Østbye, Katherine Rhodes, Gabriela Plasencia, Lauren Hart, Gregory Sawin

**Affiliations:** Department of Family Medicine & Community Health, Duke University School of Medicine, Durham, 27705, United States; Departments of Family Medicine & Community Health and Population Health Sciences, Duke University School of Medicine, Durham, 27705, United States; Department of Family Medicine & Community Health, Duke University School of Medicine, Durham, 27705, United States; Campbell University School of Osteopathic Medicine, Lillington, 27546, United States; Department of Family Medicine & Community Health and National Clinical Scholars Program, Duke University School of Medicine, Durham, 27705, United States; Department of Family Medicine & Community Health, Duke University School of Medicine, Durham, 27705, United States; Department of Family Medicine & Community Health, Duke University School of Medicine, Durham, 27705, United States

**Keywords:** medication for opioid use disorder, medication assisted treatment, opioid use disorder, opioid dependence, opioid epidemic, implementation, buprenorphine, buprenorphine/naloxone, primary care, family medicine

## Abstract

Medication for opioid use disorder (MOUD) is the management of opioid use disorder (OUD) on an outpatient basis with buprenorphine or buprenorphine/naloxone (or methadone, which is limited to federally certified opioid treatment programs). Primary care practices are well poised to provide comprehensive care for patients with OUD, including provision of MOUD. The aim of this study was to assess provider and staff OUD attitudes and role perceptions before and after implementation of a MOUD clinical service line. A survey was distributed to evaluate attitudes and perceptions of patients with OUD and provision of MOUD among providers and staff in an academic family medicine clinic. Surveys were distributed in December 2020 (73% response rate), prior to a substance use disorder educational training and MOUD service line implementation, which provided patients with OUD both primary care services and management with buprenorphine/naloxone. A follow-up survey was distributed in February 2022 (69% response rate).Training and implementation of the MOUD service line demonstrated improvements in the domains of motivation (+0.63), attitudes (+0.32), satisfaction (+0.38), role support (+0.48), role adequacy (+0.39), and safety (+0.79) among surveyed participants. The change in satisfaction and safety domains was statistically significant (*P* < .05). There was no change in the role legitimacy domain.Implementation of a primary care-based MOUD service line positively affected provider and staff motivation, attitudes, satisfaction, sense of safety, role support, and adequacy when working with patients with OUD. This highlights the benefits of MOUD-specific clinical support to optimize care delivery within primary care.

**Key messages**

- Demand continues to outpace supply for opioid addiction treatment.
- Addiction is a chronic disease well suited for management in primary care.
- Buprenorphine is an effective treatment for opioid use disorder.
- Providing addiction treatment for opioid use disorder in clinic improves worker perceptions.

## Introduction

The opioid epidemic has continued to escalate in the USA in recent years, with a staggering 79 437 opioid-related overdose deaths from July 2021 to July 2022 [1]. Even with efforts to reduce the number of circulating prescription opioids and to increase monitoring of safe prescribing practices through local, state, and federal initiatives, the number of deaths continues to rise, highlighting the importance of chronic disease management models, and primary care in the treatment of addiction.

Medication for opioid use disorder (MOUD) is the management of opioid use disorder (OUD) on an outpatient basis (also known as outpatient-based opioid treatment (OBOT)) with buprenorphine or buprenorphine/naloxone (or methadone, which is limited in use via federally certified opioid treatment programs). Despite good evidence that OBOT can reduce all-cause and opioid-related mortality [2, 3], a minority (11%) of overdose survivors receive MOUD in the first 12 months after overdose survival [4]. Buprenorphine works as well as methadone to suppress illicit opioid use when used in fixed daily doses over 2 mg [5].

Primary care providers, as experts in chronic disease management, are well positioned to provide evidence-based, patient-centered care for patients requiring MOUD. For example, a “Patient-Centered Medical Home” [6], a primary care model that emphasizes whole-person care, considers social determinants of health and the patients’ lived experiences while building a trusting continuity care team relationship. In such an approach to addiction, OUD treatment is integrated into primary care including preventive care, mental health, and comorbid chronic disease management. This can help to reduce stigma, barriers to care, and increase engagement. Though studies on PCMH interventions have mixed results, evaluations generally report improvements in preventive care, staff experience, and decreased ER visits [7].

The number of providers and clinics providing MOUD is not meeting community needs [8]. Lack of training, confidence, time, clinic and organizational support, and mental health resources have been cited as barriers to providing MOUD [9, 10]. Stigma can negatively impact patient care and experience, and concerns about patient safety can be a barrier to engagement and adoption of MOUD programs at clinic and health system levels [10]. To successfully incorporate MOUD as part of standard primary care, initiatives developing and implementing such new service lines are critical.

Recognizing the importance of MOUD in primary care, the aim of this study was to examine changes in provider and staff attitudes and role perceptions regarding working with patients with OUD from pre to postimplementation of a primary care-based MOUD clinical service line. We also tracked service line metrics to understand the impact on the practice more broadly. It was hypothesized that the incorporation of a primary care-based MOUD service line and increased familiarity and competence with aspects of OUD-specific care through substance use training and direct patient care would positively impact staff attitudes and comfort, thereby improving job motivation and satisfaction, as well as improving patient care.

## Methods

### Design

We distributed pre–post surveys to family medicine staff and providers before and after implementation of a primary care-based MOUD service line. The survey was sent to all clinic faculty and staff via email in December 2020, prior to an all-staff clinic training focused on addiction and the care of patients with OUD. The Regional Substance Use Disorder Education & Technical Assistance provider, Mountain Area Health Education Consortium delivered the two-hour training virtually [11], which all providers and staff that worked that day attended since we closed the clinic to patients to allow for full attendance. The clinic subsequently initiated a MOUD clinical service line in January 2021. We then sent a repeat survey to all clinic faculty and staff in February 2022, one year after the service line was launched to measure change in perspectives. Staff had additional interaction with the service line as they learned to navigate new MOUD appointment types and manage follow-up messages and inquiries from MOUD patients. The study was exempted from review by the Duke University Institutional Review Board.

### Study population and setting

The study was conducted at Duke Family Medicine (DFM) clinic, a university-based family medicine residency clinic that cared for 12 199 patients between January 1, 2021, and December 31, 2021 with a payor mix of 16.5% Medicaid, 21.7% Medicare, 13.8% North Carolina Blue Cross Blue Shield, 1.85% commercial, 35.9% “managed care,” and 3.5% self-pay. We distributed the survey to all clinical staff including attending and resident physicians, physician assistants, registered nurses, certified medical assistants (CMA), licensed clinical social workers (LCSW), front desk, and administrative staff. The two primary investigators were excluded from survey participation.

### Medication for opioid use disorder clinical service line

Starting in January 2021, provider-identified or self-identified family medicine patients with OUD seeking medical management with buprenorphine/naloxone were internally referred to a dedicated team comprised of two MOUD-trained family medicine providers and two LCSWs. Patients completed an initial telephone psychosocial risk assessment with an LCSW to assess for appropriateness of primary care-based MOUD along with an assessment of concurrent mental health and social needs. We provided care coordination and linkage with external mental health resources or higher levels of care as indicated.

Patients were then scheduled for consultation with dedicated MOUD-prescribing clinicians, generally within one week of initial intake, and started on buprenorphine/naloxone via home induction, with no option for observed inductions [12]. Additional topics covered during visits included mental health, peer support, medical and substance use history, overdose prevention and education, and service line expectations including a signed agreement. Patients were followed by the MOUD clinical team and received both general and preventive primary care services within the same practice.

Clinic panel data acquisition performed quarterly included monitoring total number of service line patients, indication for buprenorphine for OUD and or chronic pain management, and basic demographics. Service line metrics monitored included total number of buprenorphine/naloxone prescribing providers, level of service billed, number of no shows, and number of buprenorphine/naloxone prescriptions.

### Survey

We developed the survey to assess experience, attitudes, and perceptions regarding working with patients who have OUD across seven domains: motivation, attitudes, satisfaction, role support, role adequacy, current practice, and safety. We evaluated five domains through an adaptation of the Drugs and Drug Problems Perceptions Questionnaire (DDPPQ), a 22-item questionnaire aiming to evaluate healthcare workers role-specific therapeutic attitudes and perceptions regarding working with patients with SUD [13]. We updated DDPPQ wording to more specifically address therapeutic attitudes in regard to OUD. We removed seven items to reduce survey length and increase the likelihood of participant completion, and then re-evaluated the survey domains and considered them adequately represented by the remaining items. The safety domain was added to the survey with two items: “Patients who request treatment for opioid use disorder are more likely to become agitated or violent than average patients,” and “I worry about my safety when working with patients that have opioid use disorder.” Additional demographic information obtained included a role in the clinic, prior OUD-related experience and training, and perception of current OUD-specific care quality at the clinic and health system level. See Supplemental Material for final survey.

### Data management

We collected and managed all data electronically using Research Electronic Data Capture (REDCap) electronic data capture tools hosted by Duke University [14].

### Statistical methods

All analyses were conducted in STATA version SE/17 [15]. Since the primary aim of the study was to examine differences in provider and staff attitudes and role perceptions related to OUD before and after the implementation of a MOUD clinical service line, we examined mean differences in survey domain responses to attitudes and perceptions preand postintervention. As an additional analysis, we compared mean differences in domain responses by the provider and other roles pre and postintervention. We used linear regression to examine whether mean responses (pre or postintervention) differed for each domain. We also used linear regression to look at the mean differences in the domain by role (provider or other role) pre and postintervention. All regression models included the following variables: survey domain or attitude or perception (primary outcome), pre or postintervention (primary exposure), and provider or other role (secondary exposure).

## Results

### Medication for opioid use disorder service characteristics

**Figure 1.**
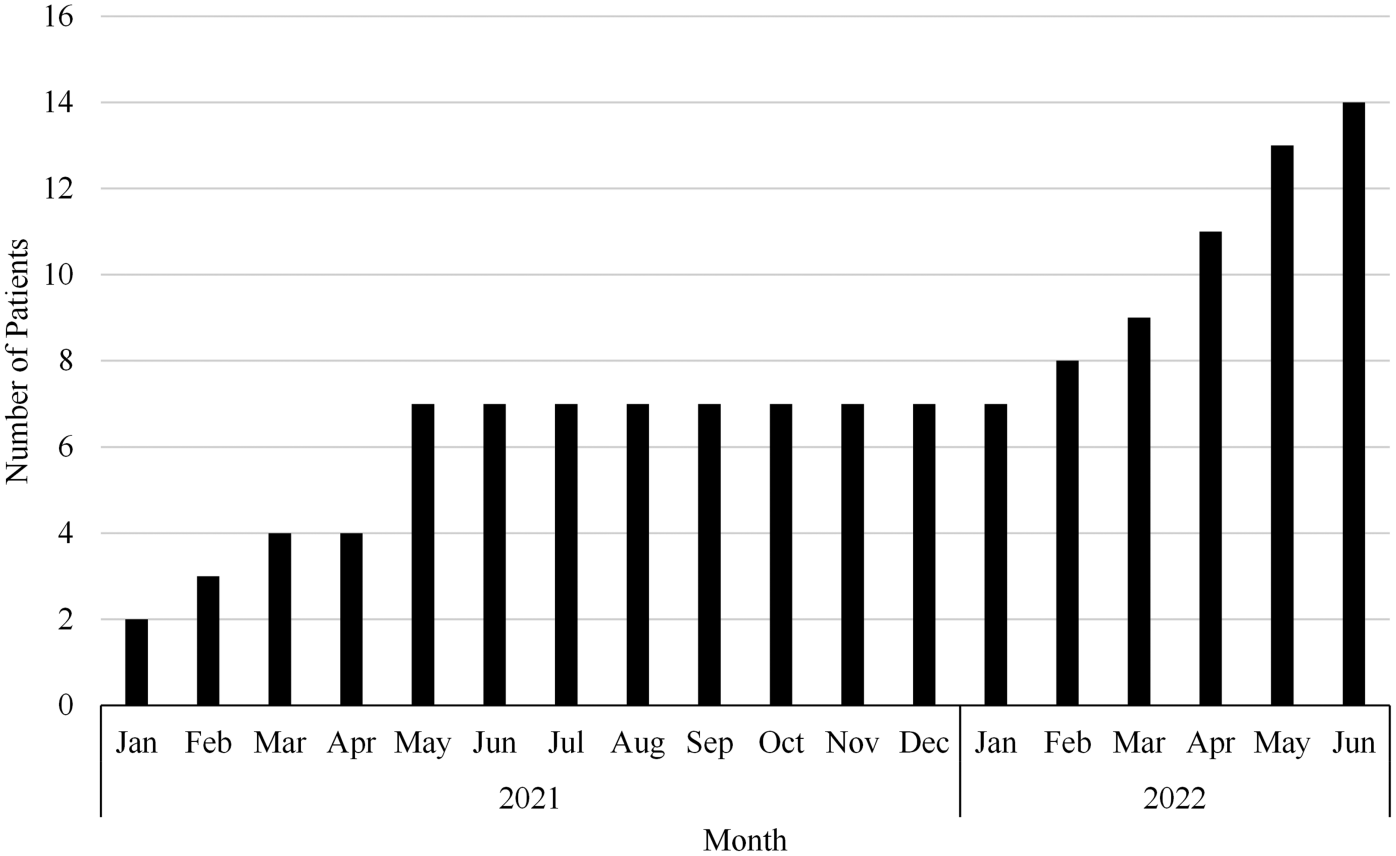
Medication for opioid use disorder clinical service line patient empanelment.

The DFM MOUD service line was initiated in January 2021 with two prescribing providers and two LCSWs acting as the core clinical team. Two additional prescribing providers were added to the clinical team in October 2021. In the subsequent year, patient empanelment increased gradually from 0 to 14 total patients with MOUD as seen in Fig. 1. As of June 2022, the panel (*n = *14) comprised 71% female patients, 29% male patients, 50% African American, 50% Caucasian, 57% between ages 18–50 and 29% older than age 65. In addition to having OUD, 50% of patients on the panel also carried a diagnosis of chronic pain. During the study period, four patients left the service line including two patients who died of unrelated illnesses, one patient who decided not to take buprenorphine/naloxone, and one patient who was lost to follow-up. DFM continued to serve as both primary care and MOUD provider for empaneled patients throughout the study period.

### Characteristics of the survey participant*s*

Of the 75 people who received the baseline survey, 55 participants completed the survey yielding a 73% overall response rate. Staff included 11 of 12 nurses (91%), 7 of 10 CMAs (70%), 7 of 9 administrative staff (77%), 2 LCSWs (100%), and 3“others.” Providers included 11 of 17 physicians (64%), 4 of 6 physician assistants (67%), 10 of 18 resident physicians (55%). We repeated the survey in February 2022 with 64 staff members, and it was completed by 44 participants yielding a 69% response rate. Staff members included 7 of 10 nurses (70%), 6 of 6 CMA (100%), 2 LCSWs (100%), and 2 of 5 administrative staff (40%). Providers included 14 of 18 physicians (77%), 9 of 18 resident physicians (50%), and 4 of 5 physician assistants (80%) (see Table 1). Of note, there was significant staff turnover in the nursing and administrative staff groups from time of baseline survey to repeat survey distribution one year later. Therefore, the participants at baseline and one year later are not identical.

**Table 1.** Participant characteristics.

	Pre		Post	
	*n*	%	*n*	%
Clinic role				
Provider*	15	27	18	40
Nurse	11	20	8	18
Resident	10	18	9	20
Medical assistant	12	22	4	9
Admin and other**	7	13	6	13
Formal training to work with patients with opioid use disorder				
Formal training	22	40	26	59
NO formal training	33	60	18	41
Waivered to be able to prescribe buprenorphine/naloxone				
Waivered	8	15	9	20
NOT waivered	17	31	18	40
Not applicable	30	55	18	40
Written a prescription for buprenorphine/naloxone				
Has written a prescription	2	4	3	7
Has NOT written a prescription	23	42	24	53
Not applicable	30	55	18	40
Cared for patients with OUD				
Has cared for patients with OUD	34	62	31	69
Has NOT cared for patients with OUD	10	18	10	22
Not applicable	11	11	4	9

*For analyses presented below, results are presented for provider and other, where other is all other clinic roles. Provider includes physicians and advanced practice professionals such as nurse practitioners and PAs.

**This category includes patient services associates, receptionists, and social workers.

### Survey results

**Figure 2.**
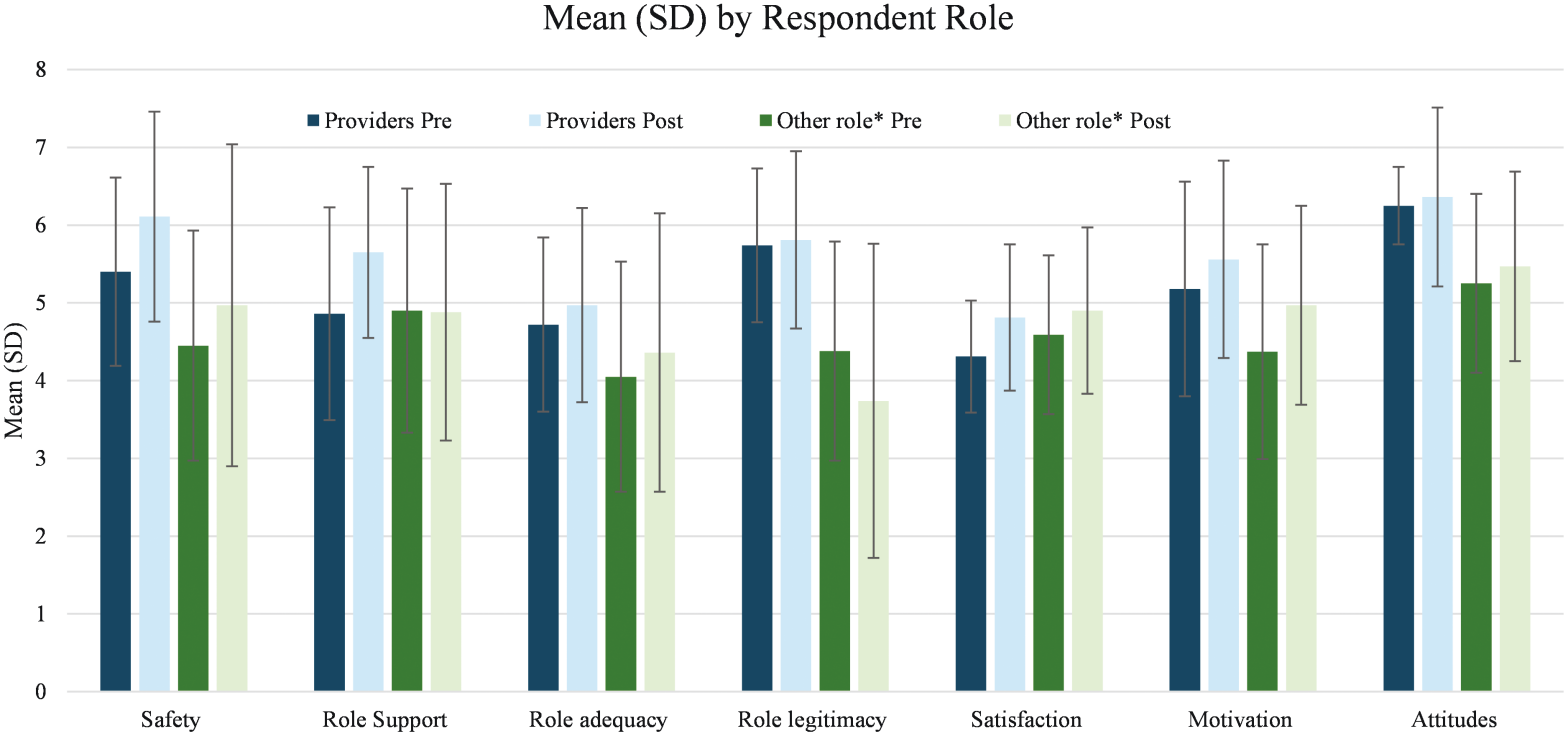
Average domain score by respondent role. *Other role = administrative, MA, nurse, social worker, other.

On completion of the follow-up survey in 2022, change in mean responses demonstrated that participants overall reported higher (more positive) responses in the domains of satisfaction (+0.38), motivation (+0.63), role adequacy (+0.39), role support (+0.48), attitudes (+0.32), and sense of safety (+0.79) in the follow-up survey as compared to the baseline survey performed prior to service line implementation in 2021 (see Fig. 2). When looking specifically at providers (physician, resident physician, physician assistant) compared to other respondents, the results were even more positive in the domains of satisfaction (+0.5), role support (+0.79), and sense of safety (+0.71). The role legitimacy domain did not change noticeably (+0.01) which comprised two survey questions: “I feel I have the right to ask patients questions about their drug use when necessary,” and “I feel that my patients believe I have the right to ask them questions about drug use when necessary.” To further evaluate this domain, responses were compared between participants with and without prior SUD-specific training. Participants with prior SUD training did report more positive role legitimacy scores as compared to participants without. Controlling for provider type, scores for all domains except for role legitimacy increased from the pre to the post survey including domains of satisfaction, motivation, role adequacy, role support, attitudes, and sense of safety. No domains worsened, and positive differences were statistically significant (*P* < .05) for satisfaction and safety (see Table 2).

**Table 2.** Linear regression analysis of domains pre- and post- intervention.

Domain	Coefficient (*β*)	95% CI	*P*
Safety	0.62	ss0.02 to 1.23	0.04
Role support	0.43	−0.16 to 1.01	0.15
Role adequacy	0.28	−0.29 to 0.85	0.34
Role legitimacy	-0.25	−0.82 to 0.31	0.37
Satisfaction	0.42	0.04 to 0.80	0.03
Motivation	0.48	−0.07 to 1.02	0.09
Attitudes	0.15	−0.27 to 0.58	0.47

All regressions were run controlling for provider type using the designations (1) provider or (2) other role.

## Discussion

### The impact of a primary care-based medication for opioid use disorder service line

While previous studies have highlighted the barriers of implementing MOUD in a primary care setting, such as training, resources, stigma, and infrastructure support [9, 10, 16], this study demonstrated that implementing MOUD care may change staff attitudes and perceptions to providing this type of care, potentially reducing concerns about acceptability and feasibility. As shown in Fig. 2., this study indicates that attitudes and perceptions regarding working with patients who have OUD improved overall with clinical exposure to a MOUD service line. Based on survey results, clinic providers and staff felt more effective and were more interested and motivated to work with patients with OUD one year after service line implementation as compared to baseline, with positive changes noted in role support, role adequacy, satisfaction, motivation, attitudes, and safety. Although the role legitimacy domain did not significantly increase from baseline to one year later, it is notable that there was a positive change in this domain when prior SUD-specific training was accounted for. This supports not only implementing MOUD, but also considering the potential value of offering more universal SUD training opportunities for staff and providers.

### Primary care medication for opioid use disorder exposure improves staff attitudes and motivation

We refer to these results as the “if you build it, they will come” effect. Due to intentional efforts to increase clinic education and build infrastructure to standardize MOUD care, the clinic saw a gradual increase in number of patients seeking addiction treatment, and also a shift in the overall culture of the clinic. Watkins et al highlight six elements that have been shown in implementation studies to improve substance use integration in primary care using similar chronic care models and collaborative development processes; delivery system redesign, self-management support, decision support, clinical information systems, health care organization and community resources [17].

Although only 14 patients were ultimately empaneled during the study period, the leadership support of the efforts, new appointment types and workflows, socialization of the care etc., increased familiarity with SUD and OUD care with persistent messaging on the logistics and importance of this work which may have impacted staff even if not directly engaged with these 14 patients. We believe this contributed to improved motivation, attitudes, satisfaction, sense of safety, role support, and adequacy. The initial goal was to introduce and pilot the service line with a small number of providers. Given the positive response observed, we have now doubled the number of buprenorphine/naloxone prescribers in clinic and are exploring how to integrate addiction care as a core competency for all family medicine residents and preceptors.

### Improving health system collaboration for medication for opioid use disorder

The findings of this study should be encouraging to practices and health systems considering ways to address the ongoing opioid epidemic. This study suggests that the implementation of an MOUD service line in primary care may improve attitudes toward patients with OUD, improve job satisfaction and motivation, and reduce stigma. Implementation of an MOUD clinical service line in a primary care setting may provide a lifeline to patients struggling with OUD in an integrated, trusted, supportive, and whole-person-oriented setting, with the potential to increase the quality of care and health-related outcomes of patients with OUD.

### Limitations

The study was limited by staff turnover in nursing and administrative staff groups during the study period. As such, the survey participants in the baseline and repeat survey groups were not identical, (see Table 1). Furthermore, new staff that joined the practice after December 2020 would not have participated in the initial SUD training in December 2020. Despite this, all providers and staff, including new hires, had exposure to patients with OUD in our clinic, whether they were directly involved with the MOUD service line or not. The subsequent 2022 survey results demonstrate net positive results (see Fig. 2). Additionally, pre and postimplementation surveys were not linked to specific participants, so we were unable to delineate progress on the individual participant level. The sample size was small due to the small size of our clinic, and this was a single-center study. Given the nature of our clinic, the results may not be generalizable. Finally, in this study, we modified the survey from a previously validated survey. The addition of our safety questions and omitting some questions for survey brevity may have altered the psychometrics properties and previous validation.

### Future research

Future research directions include further evaluation of the role legitimacy domain, including which aspects of training or patient care impact this domain. A study of patients’ perspective of their experience and impact of the implementation of the MOUD service line at our clinic could help evolve the care model in patient-centered ways. It has also been noted that 50% of the empaneled patients on the MOUD service line also carried a dual diagnosis of chronic pain. It would be beneficial to explore this patient population more closely to determine if primary care-based MOUD can beneficially impact chronic pain and quality of life from a patient perspective.

### Future practice direction

Methods to identify those at risk of addiction or with pseudoaddiction in the primary care setting would be helpful to target interventions prior to reaching diagnostic criteria for addiction or opiate use disorder. Quality of care provided to patients with OUD could be improved by creating a coalition across primary care clinics and departments in the health system involved in caring for patients with OUD to share best practices, resources, and support. Such a coalition could additionally partner with community-based organizations involved in helping patients with OUD and their families to create a community-academic coalition for greater advocacy, community impact, and systems change.

## Conclusion

Implementation of a MOUD service line and associated addiction training improved the attitudes and motivation of primary care providers and staff at a university-based family medicine residency clinic caring for patients with OUD. In the current state of a severe and unrelenting opioid crisis, we hope that this small study serves as a catalyst to propel more individuals and clinics over the activation energy of starting their own MOUD services, as we expand this life-saving evidenced-based practice more broadly across our nation.

## Supplementary data

Supplementary material is available at *Family Practice* online.

cmae044_suppl_Supplementary_Material

## Data Availability

The data underlying this article cannot be shared publicly due to concern for privacy of individuals who participated in the study. The data will be shared on reasonable request to the corresponding author.

## Prior presentations

We gave a preliminary presentation of this study after completion of the first survey and its results at the virtual NAPCRG Meeting in 2021, but have not presented or published this more complete picture of the study, which includes both the initial and follow-up survey data, demonstrating changes in perception domains. Gregory Sawin, MD, MPH; Sara Mazzarelli, MHS, PA-C; Audrey L. Blewer, PhD, MPH; Gabriela Plasencia, MD, MAS; Lauren Hart, MPH, MSW; Truls Østbye MD, MPH; Exploring barriers and facilitators for starting MAT for Opioid Use Disorder (OUD) in a Family Medicine Residency Clinic. Oral Presentation. 49th NAPCRG (North America Primary Care Research Group) Annual Meeting, November 19-23, 2021| Virtual Meeting (due to COVID-19).
