# Supplementary material for: Impact of implementing primary care-based medication for opioid use disorder on provider and staff perceptions
Source: Fam Pract. 2024 Sep 23;41(6):1018–24. doi: 10.1093/fampra/cmae044 (PMC11638083; doi:10.1093/fampra/cmae044)
Supplement: cmae044_suppl_Supplementary_Material [file cmae044_suppl_supplementary_material.pdf]

Supplementary Data. Final Study Questionnaire

1. Have you taken this survey before?
    - o Yes - I completed this survey last year
    - o No - This is my first time
    - o Not sure

2. What is your primary role in the clinic?
    - o Patient Service Associate/Receptionist/Front Desk/Clerical
    - o Medical Assistant Nurse
    - o Resident Physician
    - o APP (Physician Assistant, Nurse Practitioner) Physician
    - o Social Worker Pharmacist Other

3. At Duke Family Medicine, how effective are we in providing comprehensive care for patients that have opioid use disorder?
    - o Not effective at all
    - o Slightly effective
    - o Moderately effective
    - o Very effective Extremely effective

4. Have you cared for patients with opioid use disorder?
    - o Yes
    - o No
    - o Not applicable

5. Approximately how many patients with opioid use disorder do you see each month?
    - o 0-1
    - o 2-4
    - o 5-10
    - o More than 10

6. How easy or difficult is caring for patients with opioid use disorder? Please select the option that best describes your experience in caring for this population.
    - o Very difficult
    - o Somewhat difficult
    - o Neither easy nor difficult
    - o Somewhat easy
    - o Very easy
    - o Not applicable

7. Please indicate how much you agree or disagree with each of the following statements about working with people who have opioid use disorder.

Strongly Agree - 1	2	3	4	5	6	Strongly Disagree - 7

- I feel I have a working knowledge of drugs and drug related problems
- I feel I know enough about the physical effects of opioid dependence to carry out my role when working with patients who have opioid use disorder
- I feel I know enough about the psychological effects of opioid dependence to carry out my role when working with patients who have opioid use disorder
- I feel I can appropriately advise my patients/clients about drugs and their effects
- I feel I have the right to ask patients questions about their drug use when necessary
- I feel that my patients believe I have the right to ask them questions about drug use when necessary
- If I felt the need when working with patients who have opioid use disorder, I could easily find someone with whom I could discuss any personal difficulties that I might encounter
- If I felt the need when working with patients who have opioid use disorder, I could easily find someone who would help me clarify my professional responsibilities
- I want to work with patients who have opioid use disorder
- I feel that there is little I can do to help patients who have opioid use disorder
- In general, I have less respect for patients with opioid use disorder than for most other patients I work with
- On the whole, I am satisfied with the way I work with patients who have opioid use disorder
- In general, it is rewarding to work with patients who have opioid use disorder
- In general, I feel I can understand patients who have opioid use disorder
- Moral strength plays a large part in the development of opioid use disorder
- Treating opioid use disorder is a poor use of healthcare resources
- Patients who request treatment for opioid use disorder are more likely to become agitated or violent than average patients
- I worry about my safety when working with patients that have opioid use disorder
- As a health system (including outpatient, inpatient, emergency department), Duke Health has a coordinated approach to providing care for patients with opioid use disorder

8. Have you received any formal training to work with patients that have opioid use disorder? (examples include CME, in-service, one-on-one training)
   - Yes
   - No

9. Are you currently waivered to be able to prescribe buprenorphine/naloxone?
   - Yes
   - No

10. Have you written a prescription for buprenorphine/naloxone in the past 12 months?
    - Yes
    - No

11. Please share any additional thoughts or comments related to the care of patients with opioid misuse including opioid use disorder.
